# Integrated Analysis of MicroRNA (miRNA) and mRNA Profiles Reveals Reduced Correlation between MicroRNA and Target Gene in Cancer

**DOI:** 10.1155/2018/1972606

**Published:** 2018-12-06

**Authors:** Xingsong Li, Xiaokang Yu, Yuting He, Yuhuan Meng, Jinsheng Liang, Lizhen Huang, Hongli Du, Xueping Wang, Wanli Liu

**Affiliations:** ^1^School of Biology and Biological Engineering, Guangdong Provincial Key Laboratory of Fermentation and Enzyme Engineering, South China University of Technology, Guangzhou, China; ^2^Department of Laboratory Medicine, State Key Laboratory of Oncology in South China, Collaborative Innovation Center for Cancer Medicine, Sun Yat-sen University Cancer Center, Guangzhou, China

## Abstract

**Background:**

Accumulating evidences demonstrated that microRNA-target gene pairs were closely related to tumorigenesis and development. However, the correlation between miRNA and target gene was insufficiently understood, especially its changes between tumor and normal tissues.

**Objectives:**

The aim of this study was to evaluate the changes of correlation of miRNAs-target pairs between normal and tumor.

**Materials and Methods:**

5680 mRNA and 5740 miRNA expression profiles of 11 major human cancers were downloaded from the Cancer Genome Atlas (TCGA). The 11 cancer types were bladder urothelial carcinoma, breast invasive carcinoma, head and neck squamous cell carcinoma, kidney chromophobe, kidney renal clear cell carcinoma, kidney renal papillary cell carcinoma, liver hepatocellular carcinoma, lung adenocarcinoma, lung squamous cell carcinoma, stomach adenocarcinoma, and thyroid carcinoma. For each cancer type, we firstly obtained differentially expressed miRNAs (DEMs) and genes (DEGs) in tumor and then acquired critical miRNA-target gene pairs by combining DEMs, DEGs and two experimentally validated miRNA-target interaction databases, miRTarBase and miRecords. We collected samples with both miRNA and mRNA expression values and performed a correlation analysis by Pearson method for miRNA-target pairs in normal and tumor, respectively.

**Results:**

We totally got 4743 critical miRNA-target pairs across 11 cancer types, and 4572 of them showed weaker correlation in tumor than in normal. The average correlation coefficients of miRNA-target pairs were different greatly between normal (-0.38 ~ -0.61) and tumor (-0.04 ~ -0.26) for 11 cancer type. The pan-cancer network, which consisted of 108 edges connecting 35 miRNAs and 89 target genes, showed the interactions of pairs appeared in multicancers.

**Conclusions:**

This comprehensive analysis revealed that correlation between miRNAs and target genes was greatly reduced in tumor and these critical pairs we got were involved in cellular adhesion, proliferation, and migration. Our research could provide opportunities for investigating cancer molecular regulatory mechanism and seeking therapeutic targets.

## 1. Introduction

Cancer ranks among the most frequent causes of human death worldwide, and its incidence and mortality have risen rapidly recent years [1]. As a heterogeneous disease, cancer involves uncontrolled growth of malignant cells caused by alters of genome, methylation, mRNA, and miRNA expression [2–4]. Despite several advances being improved in diagnosis and therapy of tumor, approximate 5-year survival rate is still low [5]. Therefore, it is urgent for exploration of underlying molecules mechanisms, which would help to improve the early detection and therapy for cancer.

MicroRNAs (miRNAs) are defined as small regulatory RNA molecules with 19-24 nucleotides in length [6]. They repress gene expression by binding to complementary sequences in the 3' untranslated region (3' UTR) of mRNAs to target them for degradation and thereby prevent their translation [7]. Recent studies have demonstrated that miRNAs play an essential role in carcinogenesis, including cell migration and reproduction, by base pairing with target mRNAs of protein-coding genes [8]. Hence, screening critical miRNA-target gene pairs for cancer is a crucial work in tumor study. With the demands of miRNA-target research, the novel experimental technology like computational prediction, ChIP-seq, Microarray, and RNA sequencing had been widely used in miRNA-target identification [9], and the database for collecting experimentally validated miRNA-target pairs had been established, like miRTarBase [10] and miRecords [11]. The wide application of coexpression analysis in miRNA-target identification began with the development of Microarray and RNA sequencing [12]. With the expression values of miRNA and mRNA in multisamples, researchers could calculate correlation coefficients of miRNA-target pairs by Kendall, Spearman or Pearson method [13]. As miRNA's negatively regulation on gene expression often resulted in an inverse correlation between miRNA and its target [14], negative correlation coefficient was proposed to be an effective way of miRNA-target pair identification [15].

With the application of correlation coefficients in miRNA-target identification, miRNA-target gene pairs have consistently been suggested to play a role in tumorigenesis and progression. For example, miR-182 could result in a sustained activation of HIF1*α* pathway via targeting* PHD2* and* FIH1* in prostate cancer, which might facilitate tumor cell adaption to hypoxic stress during prostate tumor progression [16]; miR-23b, miR-365-1, and miR-365-2 were identified as biomarkers for colorectal cancer detection by exploring colorectal cancer related miRNA-target gene pairs [17]; overexpression of miR-204 reduced the gastric cancer cell proliferation by targeting* CKS1B*,* CXCL1*, and* GPRC5A* [18]. However, restricted by the number of samples, studies of correlation between miRNA and target genes in different clinical groups were still absent [19, 20]. Without a clearly understanding of correlation coefficients of miRNA-target pairs, the application of it would be limited [21]. The popularization and application of low-cost RNA sequencing, including mRNA sequencing and miRNA sequencing, gave us opportunities to measure the changes in correlation coefficients of miRNA-target pairs between different clinical groups [22].

**Figure 1:**
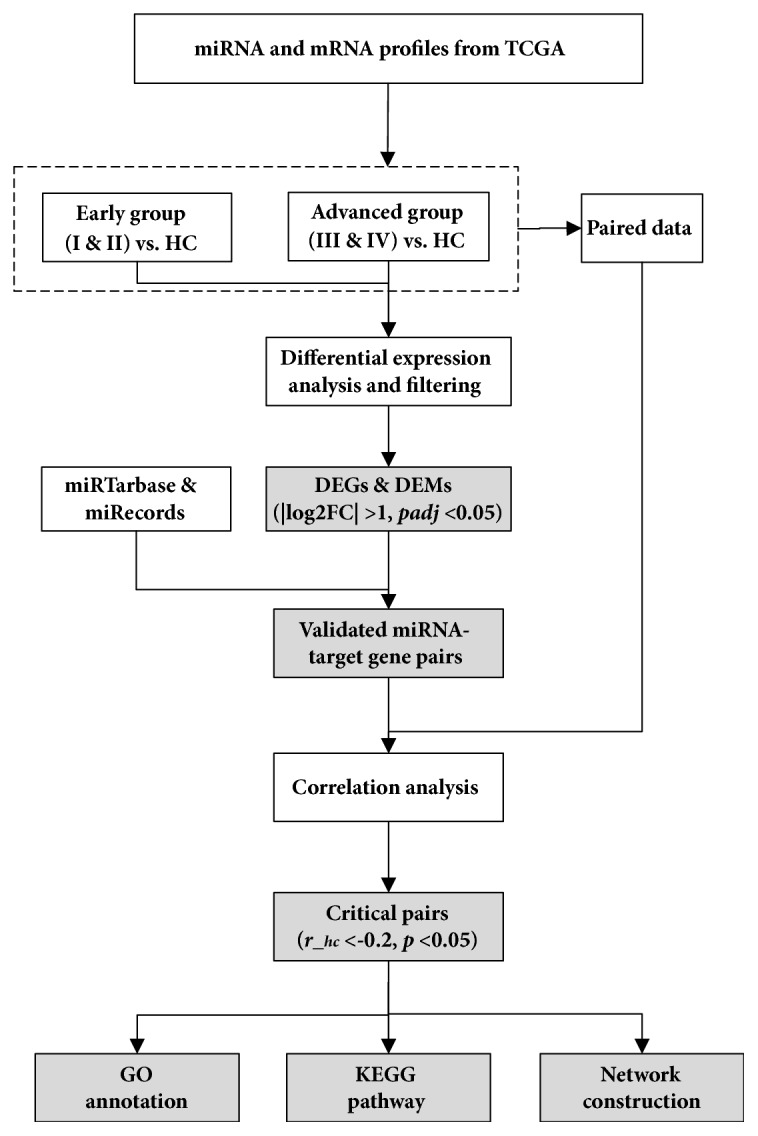
**Flowchart of this coexpression correlation analysis for miRNA-target gene pairs. **Totally 5680 RNA and 5740 miRNA sequencing samples across 11 human cancers from TCGA were included. Among these samples, 5650 samples with both miRNA and mRNA expression data were used for coexpression correlation analysis. Further, 476 patients, who donated their tumor tissues and adjacent nontumor tissues, were used to confirm the results.

The Cancer Genome Atlas (TCGA, https://cancergenome.nih.gov/) project data portal provides a platform of RNA sequencing with mRNA and miRNA for 11 000 samples across 33 different human cancer types. It contains both tumor tissue samples and adjacent normal tissue samples. Here, using the comprehensive molecular data from TCGA, we performed miRNA-target coexpression analysis and characterized the correlation changes in 11 cancer types. The workflow in this study was shown in Figure 1 and the cancer types were chosen by considering their sample sizes of healthy controls and different staging tumors, which were bladder urothelial carcinoma (BLCA), breast invasive carcinoma (BRCA), head and neck squamous cell carcinoma (HNSC), kidney chromophobe (KICH), kidney renal clear cell carcinoma (KIRC), kidney renal papillary cell carcinoma (KIRP), liver hepatocellular carcinoma (LIHC), lung adenocarcinoma (LUAD), lung squamous cell carcinoma (LUSC), stomach adenocarcinoma (STAD), and thyroid carcinoma (THCA). Our study could provide opportunities for investigating cancer molecular regulatory mechanism. The miRNA-target gene pairs would be useful as a point in further research for finding novel diagnostic biomarkers and therapeutic targets of cancer.

## 2. Materials and Methods

### 2.1. Data Resources and Preprocessing

Raw read count data of 5680 RNA sequencing samples and 5740 miRNA sequencing samples and corresponding clinical annotation files for human cancer types were downloaded from TCGA by the officially recommended GDC data transfer tool. Samples with both miRNA and mRNA expression profiles data were obtained and, further, patients who donated their tumor tissues and adjacent nontumor tissues were collected as paired samples. As a sufficient number of paired samples was important to verify the correlation analysis results, we have finally chosen 11 cancer types in this study, which were BLCA, BRCA, HNSC, KICH, KIRC, KIRP, LIHC, LUAD, LUSC, STAD, and THCA. Samples were classified into 6 groups, normal tissues, tumor tissues from stage I to IV, and tumors without stage information, according to their pathologic stages on clinical annotation files [23]. We defined normal tissues as healthy controls, stage I and stage II as early stage (Early), and stage III and stage IV as advanced stage (Advanced), whereas tumors without stage information were as TNS. The detailed sample sizes for every cancer type were presented in Table S1 and clinical information, containing sample ID, patient ID, sample stages, and groups information, was given in Table S2.

For each type of cancer, only protein coding RNA and miRNA expression data were preserved, count data were normalized by DESeq2 [24] and NormExpression were used to evaluated the normalized data quality [25]. Since the data was obtained from TCGA, further approval by an ethics committee was not required. The experimentally validated miRNA-target interactions were collected from two databases, miRTarBase (2017, Version7.0) [10] and miRecords (April 27, 2013) [11]. We integrated these two databases and merged duplicated pairs for further analysis.

### 2.2. Differential Analysis

For each cancer type, we compared expression values of miRNAs and mRNAs in early stage and advanced stage tumors as well as healthy controls by DESeq2 [24]. The thresholds were set as |log2FC| > 1 and* padj* < 0.05 to select differentially expressed miRNAs or mRNAs. miRNAs and mRNAs with log2FC > 1 were defined as upregulated, whereas those with log2FC < −1 were defined as downregulated. We removed downregulated miRNAs or mRNAs whose average expression value in healthy controls was less than 5 and upregulated miRNAs or genes whose average expression value in early or advanced was less than 5. After filtering, the low abundant miRNAs and genes were filtered out and the peaks of log values density of miRNAs and mRNAs were increased (Figure S1). Finally, we selected miRNAs and mRNAs that showed same status of regulation (upregulated or downregulated) in both early and advanced stage tumors for further analysis.

### 2.3. Critical miRNA-Target Gene Pairs Screening and Correlation Analysis

To identify critical miRNA-target gene pairs affecting tumorigenesis, we combined DEGs, DEMs and two experimentally validated miRNA-target gene databases, miRTarBase and miRecords. miRNA-target gene pairs were collected from experimentally validated interactions when both miRNA and gene were differentially expressed in tumor tissues. Samples with both miRNAs and mRNAs expression data were obtained and divided into healthy controls, early stage and advanced stage tumors. With the normalized and log2 transformed expression values, Pearson correlation coefficients of miRNA-target gene pairs were calculated for healthy controls. The pairs with Pearson correlation coefficients < -0.2 and corresponding* p*-value < 0.05 in the healthy controls were considered as tissues-specific pairs. We performed the correlation analysis for these pairs in early stage and advanced stage separately and compared the results with that in healthy controls.

### 2.4. Functional Annotation and Interaction Visualization

To gain insight into the functions of miRNA target genes, we performed Gene Ontology (GO) classification and Kyoto Encyclopedia of Genes and Genomes (KEGG) pathway enrichment analysis based on the online tools of Database for Annotation, Visualization and Integrated Discovery (DAVID) [26, 27]. Three categories were included in GO such as biological process involves pathways and cellular processes, molecular function refers to gene products activities, and cellular component to the location where gene products are active. Biological process, molecular function and cellular component. GO terms and KEGG pathways were selected using* P* < 0.05 as the cut-off. To present the regulation between miRNA and gene, critical miRNA-target interactions were used to construct miRNA-gene network by Cytoscape software [28].

### 2.5. Statistics and Figure Plotting

Statistics and calculations in this study were conducted using the software R 3.3.0 (https://www.r-project.org/) with the Bioconductor packages [29]. |log⁡2FC| > 1 and* padj* < 0.05 was considered as a statistically significant difference between values. “ggplot2” were used to visualized the figures [30].

## 3. Results

### 3.1. Differentially Expressed miRNAs and Genes in Tumor Tissues

**Figure 2:**
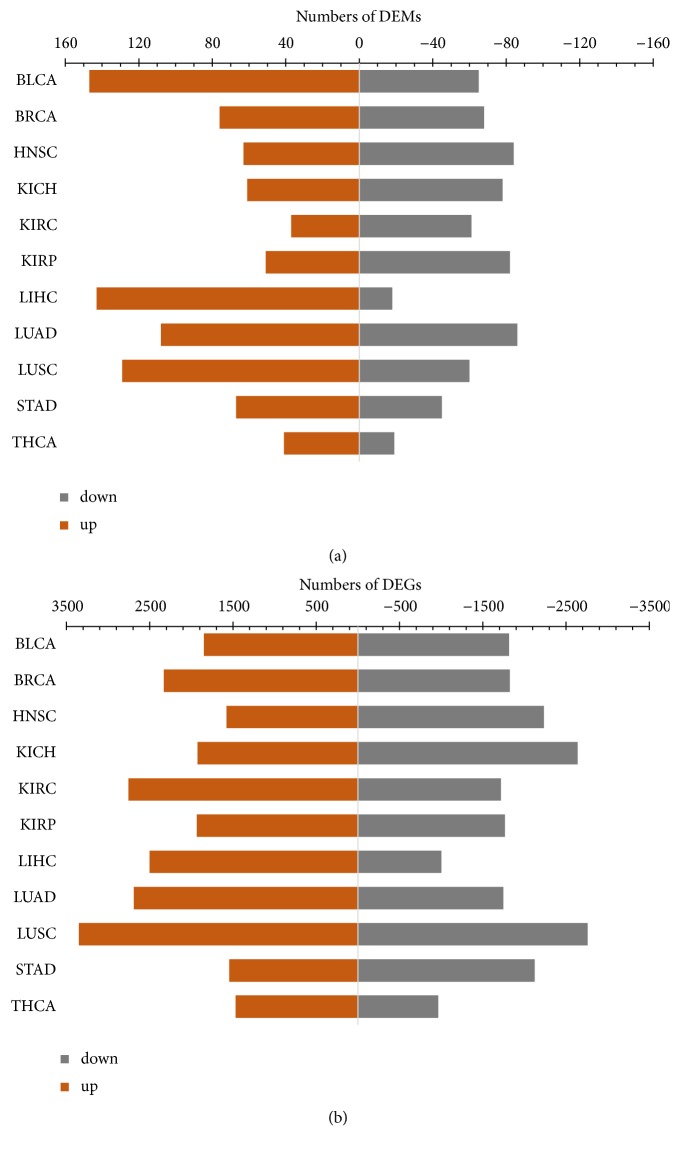
**Numbers of DEMs and DEGs in 11 cancer types. **The dark orange bars (left) denote upregulation and gray bars (right) denote downregulated mRNAs and miRNAs.

We totally identified 62 ~ 176 DEMs and 1 483 ~ 4 875 DEGs for the 11 cancer types (Figure 2). As shown in this figure, the numbers of upregulated and downregulated miRNAs varied widely in 11 cancer types. For example, 143 miRNAs were upregulated and 18 miRNAs were downregulated in LIHC; 147 miRNAs were upregulated and 65 miRNAs were downregulated in BLCA; and 129 miRNAs were upregulated and 60 miRNAs were downregulated in LUSC (Figure 2(a)). Similar phenomenon was observed for the upregulated and downregulated genes in different cancers. For example, 2 501 genes were upregulated and 1 002 genes were downregulated in LIHC, whereas, 2 754 genes were upregulated and 1 717 genes were downregulated in KIRC (Figure 2(b). These results suggested global expression differences between normal and tumor tissues. The DEMs and DEGs along with their detailed statistic items for each type of cancer were given in Tables S3 and S4 respectively.

To find out multicancer related DEGs and DEMs, we summarized DEGs and DEMs from 11 cancer types (Table S5). Totally, 12 037 DEGs and 496 DEMs were found, and 51.6% of DEMs and 77.8% of DEGs were differentially expressed in multicancers.  Table 1 listed mostly frequent DEMs and DEGs across 11 types of cancer, including genes like* ADGRB3*,* ASF1B*,* CDKN2A*,* CDT1*,* DPT*,* E2F1*,* E2F7*,* MYBL2*,* TCEAL2* and miRNAs like miR-183, miR-96, miR-1, miR-1258, miR-133a, miR-135b, miR-144, miR-182, miR-195, miR-21. These universal genes and miRNAs were mostly associated with enhanced proliferation, migration or invasion of various cancers. For example,* MYBL2* which was targeted by miR-143-3p could regulate breast cancer cell proliferation and apoptosis [31];* CDT1* was a critical regulator for normal genome replication [32], knockdown of* TGFBR3* in T24 cells could result in decreased cell growth, motility and invasion [33], and miRNAs like miR-182, miR-183, miR-21, miR-195 and miR-96 were all associated with tumorigenesis [16, 34–37]. Additionally, we identified 4 737 DEGs and 143 DEMs, which showed discordantly regulated status in different cancer types. For example,* MYBL2* was downregulated in THCA but upregulated in the remaining 10 cancer types;* FXYD1* was upregulated in KIRC but downregulated in other 10 cancer types. These results suggested the great commonness and heterogeneities among different types of tumors. The commonness could help us in finding biomarkers for multicancer, and the heterogeneities could give us guidance in individualized treatment.

### 3.2. Critical miRNA-Target Gene Pairs and Correlation Coefficients

**Figure 3:**
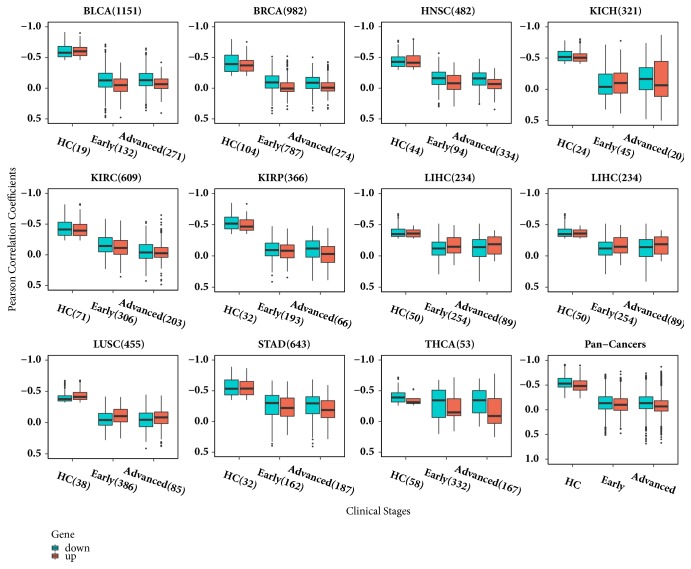
**The distribution of Pearson correlation coefficients of critical miRNA-target gene pairs in healthy controls, early stage, and advanced stage tumors.** The number next to the cancer type is the total number of critical pairs and the number next to clinical status symbol is the sample size of this group. The pairs were divided into two groups according gene's up-(red) or down-(cyan) regulated status. The pan-cancer box plots were included Pearson correlation coefficient values for healthy controls, early stage, and advanced stage tumors belonging to the 11 cancer types.

We totally found 53 ~ 1 151 critical miRNA-target gene pairs for each cancer type. The critical pairs followed with their Pearson correlation coefficients and corresponding* p* values in healthy controls, early and advanced stage tumor samples were given in Table S6. The distribution of Pearson correlation coefficients of these critical pairs in healthy controls, early and advanced stage tumors were shown in Figure 3. Obviously, the medians of Pearson correlation coefficient values of these critical pairs were higher in healthy controls than that of in early and advanced stage tumors for all 11 cancers studied. Additionally, we performed a correlation analysis for these critical pairs in samples stage I to IV separately and the results (Figure S2) still exhibited the higher Pearson correlation coefficient values in healthy controls. Additionally, we divided miRNA-target gene pairs into two groups according their up- or downregulation gene, while seldom differences were observed.

**Figure 4:**
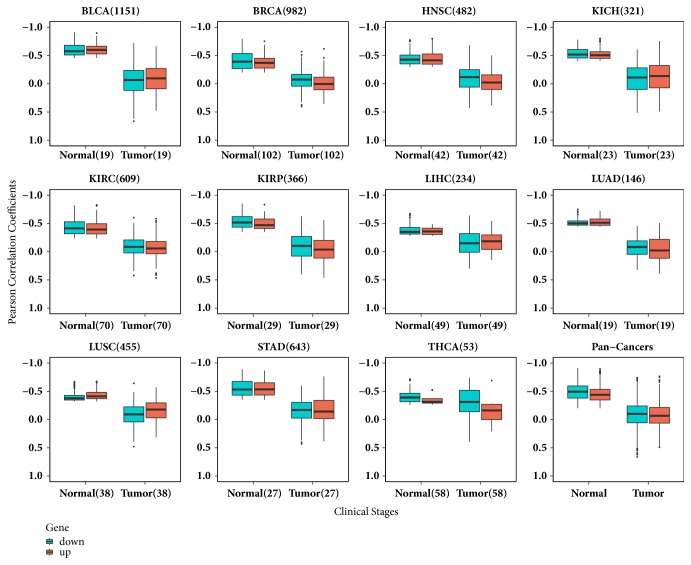
**The distribution of Pearson correlation coefficients of critical miRNA-target gene pairs in paired normal and tumor samples. **The number next to the cancer type is the total number of critical pairs and the number next to clinical status symbol is the sample size of this group. The pairs were divided into two groups according gene's up-(red) or down-(cyan) regulated status. The pan-cancer box plots include Pearson correlation coefficient values for normal and tumor belonging to the 11 cancer types.

The discrepancy in the above analysis was that the sample sizes of early and advanced stage tumors were different from those of healthy controls. Generally, the sample sizes of early or advanced were larger than those of normal, which might cause the biased results. To eliminate the impact of sample size, we found 476 patients, who not only donated their tumor tissues but also donated adjacent normal tissues, from 5650 samples. After reanalyzing, we got the correlation coefficients of miRNA-target genes which were still much higher in normal (the average Pearson correlation coefficient of -0.38 ~ -0.61) than those of in tumor (the average Pearson correlation coefficient of -0.04 ~ -0.26). The distribution of Pearson correlation coefficients of these critical pairs in normal and tumor tissues was shown in Figure 4. The above results proved that the correlation coefficient of miRNA-target pair had a difference between normal and tumor samples, and most of critical pairs showed much weaker correlation in tumor than in normal.

In order to find out universal miRNA-target gene pairs that might be associated with various cancer types, we summarized the pairs in all 11 cancer types and counted the frequency of each critical pair in 11 cancer type (Table S8). We totally got 4 743 critical miRNA-target pairs across 11 cancer types, and 4 572 pairs of them showed much weaker correlation in tumor than in normal. The most frequent pairs that appeared in at least 4 types of cancer were shown in Table 2. These common critical pairs could contribute to aberrant cell growth, survival and cell motility in various cancer types [38–40]. MiR-21 was the most common miRNA that was found in many critical miRNA-target gene pairs [37, 41].

### 3.3. Functional Annotation and miRNA-mRNA Network

We firstly identified the functions by literatures for those outstanding critical pairs in each cancer types, because these pairs showed an obviously strong correlation in normal samples but a greatly weaker correlation in tumor samples. These pairs were given in Table 3 along with their Pearson correlation coefficient values in healthy controls, early and advanced stage tumors. The experimental evidence for their target interaction and their gene functional evidence in cancer were given in Table S7. After studying the function of these genes by literature, we found these genes of these outstanding miRNA-target gene pairs were mostly involved in cell junction and cell cycle regulation. The dysregulated cell junction genes including the genes which enhance migration and invasion of cancer cells and the dysregulated cell cycle genes might enable the cells out of normal cell cycle.

To gain global insights into the biological processes regulated by the critical miRNA-target gene pairs, we performed gene ontology (GO) analysis to identify the functional categories of the target genes. The top 10 items for the molecular function, cellular component, and biological process categories of GO annotation were shown in Table S9. These included protein binding (*p* = 3.42E-05, Bonferroni correction) and cell adhesion (*p* = 1.66E-03, Bonferroni correction), which are both linked to migration of cells. The genes like* GNE*,* TRIM2*, and* PDE7A* greatly influenced cell junction [42–44]. Thus, dysregulated miRNA-target gene pairs involved in these genes like miR-21/*GNE*, miR-369/*TRIM2*, and miR-203a/*PDE7A* might promote the migration of cancer cells. Moreover, 38 critical genes in BRCA and 15 critical genes in KICH were significantly enriched in cell division (*p* = 6.29E-08, Bonferroni correction) and DNA replication cell proliferation (*p* = 5.40E-04, Bonferroni correction). The dysregulated miRNA-target gene pairs involved in these genes like miR-215/*SKA1*, miR-3653/*EMP2* and miR-192/*PIM1*, might enable the cells to evade normal constraints on cell proliferation [45–47].

We also performed KEGG pathway analysis to identify the pathways regulated by these critical pairs.  Table S10 showed 199 KEGG pathways (*p*-value < 0.05) that were enriched in the 11 cancer types. These included known cancer-related pathways, such as Pathways in cancer (*p* = 3.78E-07, Bonferroni correction, BLCA), cell cycle pathway (*p* = 1.20E-05, Bonferroni correction, BRCA), p53 signaling pathway (*p* = 8.83E-06, Bonferroni correction, HNSC), and AMPK signaling pathway (*p* = 3.60E-04, Bonferroni correction, KIRC). The dysregulated miRNA-target gene pairs involved these pathways might modulate the development and progression of tumors. The most frequently dysregulated pathways were listed in Table 4. Particularly, the term ‘Cell cycle' was included in 8 cancer types (BLCA, BRCA, HNSC, KIRC, KIRP, LUAD, LUSC, STAD) and ‘pathways in cancer' was included in 6 cancer types (BLCA, BRCA, KIRP, LUAD, STAD, THCA).

**Figure 5:**
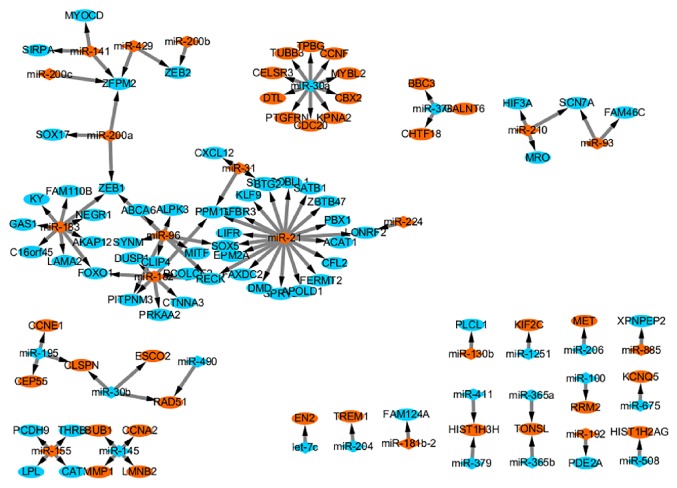
**Pan-cancer miRNA-target gene network. **The diamond nodes represent miRNAs; the circle nodes represent target genes; red and blue colors indicate downregulation and upregulation, respectively; solid lines denote association between the miRNAs and the target genes.

For a better visualization of these miRNA-target gene interactions, we constructed the miRNA-mRNA network by using miRNA-target gene interaction in Table S6. Common miRNA-target pairs were usually deserved more attention because their universality in multicancer types. We constructed a pan-cancer network with the miRNA-target gene pairs that appear in at least three cancer types (Figure 5). The network consisted of 108 edges connecting 35 miRNAs and 89 target genes. Among these, miR-21 and miR-30a were the most common regulators, which were linked to 20 and 10 target genes, respectively.

## 4. Discussion

The correlation coefficient, like Pearson, Kendall, Spearman method correlation coefficient, was widely used to measure the intensity of association between two variables [48]. miRNA's negative regulation on gene expression could result in an inverse correlation between miRNA and its target, so negatively correlation coefficient was proposed to be an effective way of miRNA-target pair identification [49], while seldom study analyzed the changes of miRNA–mRNA interactions in tumor and normal [22]. Our data suggested that the negative correlation of several critical miRNA-target gene pairs had a global reduction in tumor tissues compared with in normal. And these correlation reduced critical pairs were mostly participant in tumorigeneses and tumor progression.

Since the expression changes of miRNA or mRNA regulated by various factors could cause the changes of correlation between them, the factors who result in the reduced correlation in tumor tissues were indistinct. We speculated that correlation regulatory networks of these critical pairs were rewired in cancer cells by following reasons. Firstly, decreased expression of core miRNA in tumor tissues might diminish the negative regulation of its target genes. Only the most abundantly expressed miRNAs could affect their target mRNAs stability by effectively binding to the available mRNA target sites [50]. Secondly, the miRNA-target gene pairs had specific interact manner due to particular vivo environmental [51]. The condition-specific regulation of miRNA might cause different correlation of miRNA-target pair in tumor and normal tissues. Thirdly, changes in the intracellular environment, especially cellular metabolism, might reduce the correlation of miRNA-target gene pairs. Highly proliferating cancer cells showed profound metabolic changes in response to the increased demands of energy, reducing power and building blocks to sustain their elevated biosynthetic and physiological needs [52]. The Warburg effect indicated that cancer cells increase glycolysis and alter the intracellular acid-base balance in cancer cells [53], which might not conducive to miRNA binding its target mRNAs.

Moreover, transcription factors [54] and endogenous long noncoding RNAs [55] might also affect the correlation of miRNA-target gene pair. Whole gene methylation and gene copy number were significantly different between tumor and normal, which have been confirmed to be closely related to gene and miRNA expression [56]. Cause of variation of pairs correlation was so complex, there was a need for systematic study. Several previous studies had measured the correlation between miRNA and mRNA expression in tumor samples using a multivariate linear model that eliminate the noise of copy number alters and methylation changes [57, 58]. Based on their method, we estimated the effect on correlation of miRNA-target gene pairs by methylation and copy number altersing. The detailed processes were given in the Supplementary Material, and the results were shown in Figures S3–S6 and Table S11. From these results, we found that methylation and copy number altering really affected the correlation of miRNA-target pairs but had no significance. This conclusion was consistent with results in Jacobsen's and Andres-Leon's researches. For example, the correlation coefficient of miR-20b/*RORC* in Jacobsen's work just increased from -0.08 to -0.20. And the average correlation coefficients of pairs in Andres-Leon E et al. work were similar to ours (both close to -0.1), which were all much smaller than the average correlation coefficient values of the pairs in normal tissue (nearly to -0.5).

## 5. Conclusion

In summary, this integrative analysis had confirmed the changed correlation of miRNA-target gene pairs in different clinical status and the reduced correlation of several critical pairs in tumor compared with in normal which was not observed in previous studies. These critical pairs were found to be mostly participant in biological processes and signaling pathways, such as focal adhesion, p53, MAPK, and PI3K-Akt signaling pathways, who were hubs pathways in cancer progression, invasion, and metastasis. This study had presented the molecular regulatory mechanism and the critical pairs would be useful for finding novel diagnostic biomarkers and therapeutic targets of cancer.

## Figures and Tables

**Table 1:** The most common DEGs and DEMs in 11 cancer types.

**DEGs or DEMs**	**BLCA**	**BRCA**	**HNSC**	**KICH**	**KIRC**	**KIRP**	**LIHC**	**LUAD**	**LUSC**	**STAD**	**THCA**	**Frequency**
*ADGRB3*	down	down	down	down	down	down	down	down	down	down	up	11
*ASF1B*	up	up	up	up	up	up	up	up	up	up	up	11
*CDKN2A*	up	up	up	up	up	up	up	up	up	up	up	11
*CDT1*	up	up	up	up	up	up	up	up	up	up	up	11
*DPT*	down	down	down	down	down	down	down	down	down	down	down	11
*E2F1 *&* E2F7*	up	up	up	up	up	up	up	up	up	up	up	11
*MYBL2*	up	up	up	up	up	up	up	up	up	up	down	11
*TCEAL2*	down	down	down	down	down	down	down	down	down	down	down	11
*TGFBR3*	down	down	down	down	down	down	down	down	down	down	down	11

miR-183	up	up	up	up	/	up	up	up	up	up	up	10
miR-96	up	up	up	up	/	up	up	up	up	up	up	10
miR-1	down	down	down	down	down	down	/	down	down	down	/	9
miR-1258	down	down	down	up	/	/	down	down	down	down	down	9
miR-133a	down	down	down	down	down	down	/	down	down	down	/	9
miR-135b	up	up	up	down	/	up	up	up	up	up	/	9
miR-144	/	down	down	up	up	up	/	down	down	down	down	9
miR-182	up	up	/	up	/	up	up	up	up	up	up	9
miR-195	down	down	down	down	/	down	down	down	down	down	/	9
miR-21	up	up	up	/	up	up	up	up	/	up	up	9

**Note. **‘up' denotes upregulated; ‘down' denotes downregulated; ‘/'denotes no differentially expressed.

**Table 2:** The most common critical miRNA-target gene pairs in 11 cancer types.

Critical pairs	BLCA	BRCA	HNSC	KICH	KIRC	KIRP	LIHC	LUAD	LUSC	STAD	THCA	Frequency
miR-183/*NEGR1*	0.64	0.50	/	0.31	/	0.18	/	/	/	0.50	0.25	6
miR-210/*SCN7A*	0.16	-0.37	0.20	/	0.31	0.49	/	/	/	0.00	/	6
miR-21/*EPM2A*	/	0.34	0.65	/	0.23	0.30	0.03	/	/	0.49	/	6
miR-21/*LIFR*	0.42	0.47	0.62	/	/	0.32	/	/	/	/	-0.05	5
miR-182/*PCOLCE2*	0.51	0.30	/	0.68	/	0.42	/	/	/	0.30	/	5
miR-21/*FAXDC2*	/	0.57	0.74	/	/	/	0.04	/	/	0.47	-0.32	5
miR-21/*SOX5*	0.16	0.66	/	/	/	/	/	/	/	0.24	0.43	4
miR-183/*AKAP12*	0.40	0.43	/	/	/	/	/	/	/	0.77	0.05	4
miR-195/*CCNE1*	0.16	-0.13	0.08	/	/	/	/	0.25	/	/	/	4
miR-96/*RECK*	0.63	0.23	0.45	/	/	/	/	/	/	0.19	/	4

**Note: **The value equals Pearson correlation coefficient value in tumor group minus Pearson correlation coefficient value in normal group. Larger value means greater difference of correlation for the miRNA-target gene pairs in the tumor tissues relative to normal tissues. ‘/' denotes absence of this pair in this cancer type.

**Table 3:** The top two miRNA-target gene pairs in healthy controls.

Critical pairs	Cancer	*r*_*_hc*_ (*p*-value)	*r*_*_early*_ (*p*-value)	*r*_*_advanced*_ (*p*-value)
miR-429/*KLHL42*	BLCA	-0.91(5.34E-08)	-0.20(2.11E-02)	-0.16(8.89E-03)
miR-200c/*KLHL42*	BLCA	-0.91(5.74E-08)	-0.22(1.28E-02)	-0.14(2.00E-02)
miR-200a/*HHIP*	BRCA	-0.80(2.74E-24)	-0.09(1.68E-02)	0.04(4.75E-01)
miR-200b/*HHIP*	BRCA	-0.80(3.34E-24)	-0.08(2.46E-02)	0.02(7.42E-01)
miR-30a/*CDC20*	HNSC	-0.80(8.01E-11)	-0.17(1.09E-01)	-0.02(7.76E-01)
miR-30a/*KPNA2*	HNSC	-0.80(8.75E-11)	-0.21(3.97E-02)	-0.15(7.20E-03)
miR-369/*TRIM2*	KICH	-0.80(2.26E-06)	-0.25(9.14E-02)	-0.62(3.60E-03)
miR-127/*TMEM116*	KICH	-0.79(4.63E-06)	-0.21(1.64E-01)	-0.66(1.69E-03)
miR-203a/*PDE7A*	KIRC	-0.83(7.19E-19)	-0.02(7.12E-01)	0.06(3.61E-01)
miR-203a/*SPATA18*	KIRC	-0.82(1.54E-18)	0.01(8.50E-01)	0.11(1.24E-01)
miR-31/*SLC16A9*	KIRP	-0.85(7.81E-10)	0.20(5.96E-03)	0.06(6.26E-01)
miR-589/*FGF1*	KIRP	-0.85(1.01E-09)	-0.16(2.42E-02)	0.04(6.26E-01)
miR-21/*EPM2A*	LIHC	-0.67(9.03E-08)	-0.45(8.85E-14)	-0.24(2.07E-02)
miR-21/*GNE*	LIHC	-0.67(1.06E-07)	-0.24(9.07E-05)	-0.24(2.51E-02)
miR-493/*AGTPBP1*	LUAD	-0.75(1.49E-04)	-0.17(5.89E-04)	-0.17(8.77E-02)
let-7d/*CLDN12*	LUAD	-0.72(2.99E-04)	0.04(4.07E-01)	0.08(4.18E-01)
miR-30d*/MYBL2*	LUSC	-0.68(3.19E-06)	-0.22(1.96E-05)	-0.10(3.71E-01)
miR-30b/*CELSR3*	LUSC	-0.67(3.49E-06)	0.06(2.32E-01)	0.06(6.15E-01)
miR-200a/*TCF7L1*	STAD	-0.89(1.06E-11)	-0.59(8.29E-17)	-0.42(2.71E-09)
miR-183/*NEGR1*	STAD	-0.89(1.11E-11)	-0.59(1.96E-16)	-0.50(5.31E-13)
miR-221/*PRDM16*	THCA	-0.72(2.56E-10)	-0.41(5.85E-15)	-0.41(3.41E-08)
miR-221/*ASXL3*	THCA	-0.71(3.01E-10)	-0.50(9.48E-23)	-0.53(2.19E-13)

**Note: **Pearson correlation coefficients and its adjusted *p*-value are listed. These miRNA-target gene pairs showed stronger correlation in healthy controls than in early stage and advanced stage tumors.

**Table 4:** List of the enriched KEGG pathways in 11 cancer types.

TermID	TermName	CancerType	Frequency
hsa04110	Cell cycle	BLCA; BRCA; HNSC; KIRC; KIRP; LUAD; LUSC; STAD	8
hsa05200	Pathways in cancer	BLCA; BRCA; KIRP; LUAD; STAD; THCA	6
hsa00071	Fatty acid degradation	HNSC; KIRC; KIRP; LIHC	4
hsa04020	Calcium signaling pathway	BLCA; KIRP; LIHC; STAD	4
hsa04114	Oocyte meiosis	BLCA; BRCA; HNSC; KIRP	4
hsa04115	p53 signaling pathway	BRCA; HNSC; LUSC; STAD	4
hsa04510	Focal adhesion	BLCA; BRCA; KIRC; LUAD	4
hsa05205	Proteoglycans in cancer	BLCA; BRCA; KIRC; LIHC	4
hsa05414	Dilated cardiomyopathy	BLCA; BRCA; HNSC; KICH	4
hsa04024	cAMP signaling pathway	BLCA; KICH; STAD	3
hsa04068	FoxO signaling pathway	BLCA; BRCA; KIRP	3
hsa04151	PI3K-Akt signaling pathway	BLCA; KIRC; LUAD	3
hsa04512	ECM-receptor interaction	BLCA; LUAD; LUSC	3
hsa04550	Signaling pathways regulating pluripotency of stem cells	BLCA; BRCA; LIHC	3
hsa04914	Progesterone-mediated oocyte maturation	BLCA; BRCA; HNSC	3
hsa05161	Hepatitis B	BLCA; KIRC; STAD	3
hsa05202	Transcriptional misregulation in cancer	HNSC; KIRC; STAD	3
hsa05410	Hypertrophic cardiomyopathy (HCM)	BLCA; BRCA; HNSC	3
hsa05166	HTLV-I infection	BRCA; KIRC; LUSC	3
hsa04931	Insulin resistance	BLCA; BRCA; KIRP	3

## Data Availability

The data used to support the findings of this study are available from the Cancer Genome Atlas freely.

## Abbreviations

DEM: Differentially expressed miRNA
DEG: Differentially expressed gene
BLCA: Bladder urothelial carcinoma
BRCA: Breast invasive carcinoma
HNSC: Head and neck squamous cell carcinoma
KICH: Kidney chromophobe
KIRC: Kidney renal clear cell carcinoma
KIRP: Kidney renal papillary cell carcinoma
LIHC: Liver hepatocellular carcinoma
LUAD: Lung adenocarcinoma
LUSC: Lung squamous cell carcinoma
STAD: Stomach adenocarcinoma
THCA: Thyroid carcinoma.

## Supplementary Materials

Supplementary Materials Figure S1. Expression density distribution of mRNAs and miRNAs before and after filtering. Figure S2. Distribution of the PCC values of critical miRNA-target gene pairs in healthy controls and 4 clinically staged tumors. Figure S3: Simple correlations coefficients (Cor) and partial correlation coefficients (Pcor) of critical pairs in normal and tumor samples of LIHC. Figure S4: Simple correlations coefficients (Cor) and partial correlation coefficients (Pcor) of critical pairs in normal and tumor samples of LUAD. Figure S5: Simple correlations coefficients (Cor) and partial correlation coefficients (Pcor) of critical pairs in tumor samples of LIHC. Figure S6: Simple correlations coefficients (Pearson correlation coefficient) and partial correlation coefficients (given CNAs effects) of critical pairs in cancer samples of LUAD. Table S1: Sample sizes for each cancer type. Table S2. Sample clinical and grouping information for each cancer type. Table S3. List of DEMs for each cancer type. Table S4. List of DEGs for each cancer type. Table S5. Frequency of DEMs and DEGs in 11 cancer types. Table S6. List of critical miRNA-target gene pairs for each cancer type. Table S7: Interactional references and gene functional references for some critical miRNA-target pairs. Table S8. Frequency of critical miRNA-target pairs in 11 cancer types. Table S9. List of the significantly GO annotation terms (p < 0.05) for the critical target genes in each cancer type. Table S10. List of the significantly KEGG pathway terms (p < 0.05) for the critical target genes in each cancer type. Table S11. Partial and simple correlation coefficients for each critical pair in normal and tumor samples of LUAD and LIHC when controlling methylation or CNAs effect.
