# Supplementary material for: Integrated Analysis of MicroRNA (miRNA) and mRNA Profiles Reveals Reduced Correlation between MicroRNA and Target Gene in Cancer
Source: Biomed Res Int. 2018 Dec 6;2018:1972606. doi: 10.1155/2018/1972606 (PMC6304515; doi:10.1155/2018/1972606)
Supplement: Supplementary Materials — Figure S1. Expression density distribution of mRNAs and miRNAs before and after filtering. Figure S2. Distribution of the PCC values of critical miRNA-target gene pairs in healthy controls and 4 clinically staged tumors. Figure S3: Simple correlations coefficients (Cor) and partial correlation coefficients (Pcor) of critical pairs in normal and tumor samples of LIHC. Figure S4: Simple correlations coefficients (Cor) and partial correlation coefficients (Pcor) of critical pairs in normal and tumor samples of LUAD. Figure S5: Simple correlations coefficients (Cor) and partial correlation coefficients (Pcor) of critical pairs in tumor samples of LIHC. Figure S6: Simple correlations coefficients (Pearson correlation coefficient) and partial correlation coefficients (given CNAs effects) of critical pairs in cancer samples of LUAD. Table S1: Sample sizes for each cancer type. Table S2. Sample clinical and grouping information for each cancer type. Table S3. List of DEMs for each cancer type. Table S4. List of DEGs for each cancer type. Table S5. Frequency of DEMs and DEGs in 11 cancer types. Table S6. List of critical miRNA-target gene pairs for each cancer type. Table S7: Interactional references and gene functional references for some critical miRNA-target pairs. Table S8. Frequency of critical miRNA-target pairs in 11 cancer types. Table S9. List of the significantly GO annotation terms (p < 0.05) for the critical target genes in each cancer type. Table S10. List of the significantly KEGG pathway terms (p < 0.05) for the critical target genes in each cancer type. Table S11. Partial and simple correlation coefficients for each critical pair in normal and tumor samples of LUAD and LIHC when controlling methylation or CNAs effect. [file 1972606.f1.zip › 1972606.f1/Table S1_BMRI_2598510.docx]

## Table S1: Detail information of sample sizes in 11 cancer types.

| **Steps** | **Step 1 (Differential analysis )** | | | | | | | | **Step 2 (Correlation analysis)** | | | | **Step 3 (Confirm correlation result)** | |
| --- | --- | --- | --- | --- | --- | --- | --- | --- | --- | --- | --- | --- | --- | --- |
| **Cancer**  **type** | **RNA-seq** | | | | **miRNA-seq** | | | | **RNA-seq and miRNA-seq** | | | | **Paired samples** | |
|  | **HC** | **Early** | **Advanced** | **TNS** | **HC** | **Early** | **Advanced** | **TNS** | **HC** | **Early** | **Advanced** | **TNS** | **Normal** | **Tumor** |
| **BLCA** | 19 | 132 | 274 | 2 | 19 | 133 | 274 | 2 | 19 | 132 | 271 | 2 | 19 | 19 |
| **BRCA** | 113 | 800 | 279 | 11 | 104 | 790 | 277 | 10 | 104 | 787 | 274 | 10 | 102 | 102 |
| **HNSC** | 44 | 95 | 337 | 68 | 44 | 100 | 349 | 74 | 44 | 94 | 334 | 67 | 42 | 42 |
| **KICH** | 24 | 45 | 20 | 0 | 25 | 46 | 20 | 0 | 24 | 45 | 20 | 0 | 23 | 23 |
| **KIRC** | 72 | 322 | 205 | 3 | 71 | 308 | 205 | 3 | 71 | 306 | 203 | 3 | 70 | 70 |
| **KIRP** | 29 | 193 | 66 | 29 | 31 | 194 | 67 | 30 | 32 | 193 | 66 | 29 | 29 | 29 |
| **LIHC** | 50 | 257 | 90 | 24 | 50 | 258 | 90 | 24 | 50 | 254 | 89 | 24 | 49 | 49 |
| **LUAD** | 49 | 395 | 110 | 8 | 46 | 398 | 108 | 7 | 20 | 392 | 108 | 7 | 19 | 19 |
| **LUSC** | 49 | 406 | 91 | 4 | 45 | 388 | 86 | 4 | 38 | 386 | 85 | 4 | 38 | 38 |
| **STAD** | 32 | 164 | 188 | 23 | 41 | 186 | 223 | 27 | 32 | 162 | 187 | 23 | 27 | 27 |
| **THCA** | 58 | 333 | 167 | 2 | 59 | 336 | 186 | 2 | 58 | 332 | 167 | 2 | 58 | 58 |
| **Sum** | 539 | 3042 | 1827 | 174 | 535 | 3137 | 1885 | 183 | 492 | 3083 | 1904 | 171 | 476 | 476 |
| **Total** | 5680 | | | | 5740 | | | | 5650 | | | | 952 | |

**HC:** Health control tissues samples; **Early**: Stage I and Stage II Tumor tissues samples; **Advanced:** Stage III and Stage IV Tumor tissues samples; **TNS;** Tumor tissues with No stage information; **Patients;** People who volunteer to donate their tumor tissues and adjacent non-tumor tissues samples; **Normal:** Adjacent non-tumor somatic tissues samples of Patients; **Tumor:** Tumor tissues of Patients.
