# Supplementary material for: Integrated Analysis of MicroRNA (miRNA) and mRNA Profiles Reveals Reduced Correlation between MicroRNA and Target Gene in Cancer
Source: Biomed Res Int. 2018 Dec 6;2018:1972606. doi: 10.1155/2018/1972606 (PMC6304515; doi:10.1155/2018/1972606)
Supplement: Supplementary Materials — Figure S1. Expression density distribution of mRNAs and miRNAs before and after filtering. Figure S2. Distribution of the PCC values of critical miRNA-target gene pairs in healthy controls and 4 clinically staged tumors. Figure S3: Simple correlations coefficients (Cor) and partial correlation coefficients (Pcor) of critical pairs in normal and tumor samples of LIHC. Figure S4: Simple correlations coefficients (Cor) and partial correlation coefficients (Pcor) of critical pairs in normal and tumor samples of LUAD. Figure S5: Simple correlations coefficients (Cor) and partial correlation coefficients (Pcor) of critical pairs in tumor samples of LIHC. Figure S6: Simple correlations coefficients (Pearson correlation coefficient) and partial correlation coefficients (given CNAs effects) of critical pairs in cancer samples of LUAD. Table S1: Sample sizes for each cancer type. Table S2. Sample clinical and grouping information for each cancer type. Table S3. List of DEMs for each cancer type. Table S4. List of DEGs for each cancer type. Table S5. Frequency of DEMs and DEGs in 11 cancer types. Table S6. List of critical miRNA-target gene pairs for each cancer type. Table S7: Interactional references and gene functional references for some critical miRNA-target pairs. Table S8. Frequency of critical miRNA-target pairs in 11 cancer types. Table S9. List of the significantly GO annotation terms (p < 0.05) for the critical target genes in each cancer type. Table S10. List of the significantly KEGG pathway terms (p < 0.05) for the critical target genes in each cancer type. Table S11. Partial and simple correlation coefficients for each critical pair in normal and tumor samples of LUAD and LIHC when controlling methylation or CNAs effect. [file 1972606.f1.zip › 1972606.f1/Table S7_BMRI_2598516.docx]

## Table S7: Interactional references and Gene functional references for some critical miRNA-target pairs.

| **miRNAs** | **Genes** | **CancerTypes** | **Experimental evidence for target interaction (PMID)** | **Gene functional evidence in cancer** |
| --- | --- | --- | --- | --- |
| miR-429 | *KLHL42* | BLCA | 23446348 | [1] |
| miR-200c | *KLHL42* | BLCA | 23446348 | [1] |
| miR-200a | *HHIP* | BRCA | 21572407 | [2] |
| miR-200b | *HHIP* | BRCA | 21572407 | [2] |
| miR-30a | *CDC20* | HNSC | 18668040 | [3] |
| miR-30a | *KPNA2* | HNSC | 24398324 | [4] |
| miR-369 | *TRIM2* | KICH | 23446348 | [5] |
| miR-127 | *TMEM116* | KICH | 24374217 | [6] |
| miR-203a | *PDE7A* | KIRC | 22100165 | [7] |
| miR-203a | *SPATA18* | KIRC | 21572407 | [8] |
| miR-31 | *SLC16A9* | KIRP | 22100165 | [9] |
| miR-589 | *FGF1* | KIRP | 19536157 | [10] |
| miR-21 | *EPM2A* | LIHC | 18591254 | [11] |
| miR-21 | *GNE* | LIHC | 18591254 | [12] |
| miR-493 | *AGTPBP1* | LUAD | 24374217 | NA |
| let-7d | *CLDN12* | LUAD | 24398324 | [13] |
| miR-30d | *MYBL2* | LUSC | 22473208 | [14] |
| miR-30b | *CELSR3* | LUSC | 20371350 | [15] |
| miR-200a | *TCF7L1* | STAD | 20005803 | [16] |
| miR-183 | *NEGR1* | STAD | 23824327 | [17] |
| miR-221 | *PRDM16* | THCA | 23622248 | [18] |
| miR-221 | *ASXL3* | THCA | 20371350 | [19] |

1. Cummings, C.M., et al., *The Cul3/Klhdc5 E3 ligase regulates p60/katanin and is required for normal mitosis in mammalian cells.* J Biol Chem, 2009. **284**(17): p. 11663-75.

2. Xu, J., et al., *Correlation between lung cancer and the HHIP polymorphisms of chronic obstructive pulmonary disease (COPD) in the Chinese Han population.* Genes Immun, 2018.

3. Parmar, M.B., et al., *Additive Polyplexes to Undertake siRNA Therapy against CDC20 and Survivin in Breast Cancer Cells.* Biomacromolecules, 2018.

4. Li, J., et al., *KPNA2 promotes metabolic reprogramming in glioblastomas by regulation of c-myc.* J Exp Clin Cancer Res, 2018. **37**(1): p. 194.

5. Qin, Y., et al., *TRIM2 regulates the development and metastasis of tumorous cells of osteosarcoma.* Int J Oncol, 2018. **53**(4): p. 1643-1656.

6. Wrzesinski, T., et al., *Expression of pre-selected TMEMs with predicted ER localization as potential classifiers of ccRCC tumors.* BMC Cancer, 2015. **15**: p. 518.

7. Yamamoto, N., et al., *The tumor-suppressive microRNA-1/133a cluster targets PDE7A and inhibits cancer cell migration and invasion in endometrial cancer.* Int J Oncol, 2015. **47**(1): p. 325-34.

8. Bornstein, C., et al., *SPATA18, a spermatogenesis-associated gene, is a novel transcriptional target of p53 and p63.* Mol Cell Biol, 2011. **31**(8): p. 1679-89.

9. Fernandez-Ranvier, G.G., et al., *Identification of biomarkers of adrenocortical carcinoma using genomewide gene expression profiling.* Arch Surg, 2008. **143**(9): p. 841-6; discussion 846.

10. Wu, D., et al., *Upregulation of long non-coding RNA RAB1A-2 induces FGF1 expression worsening lung cancer prognosis.* Cancer Lett, 2018. **438**: p. 116-125.

11. Sun, Y., et al., *Screening of Differently Expressed miRNA and mRNA in Prostate Cancer by Integrated Analysis of Transcription Data.* Urology, 2016. **94**: p. 313.e1-6.

12. Kemmner, W., et al., *Loss of UDP-N-acetylglucosamine 2-epimerase/N-acetylmannosamine kinase (GNE) induces apoptotic processes in pancreatic carcinoma cells.* Faseb j, 2012. **26**(2): p. 938-46.

13. Hansen, S.N., et al., *The stepwise evolution of the exome during acquisition of docetaxel resistance in breast cancer cells.* BMC Genomics, 2016. **17**: p. 442.

14. Guan, Z., et al., *High MYBL2 expression and transcription regulatory activity is associated with poor overall survival in patients with hepatocellular carcinoma.* Curr Res Transl Med, 2018. **66**(1): p. 27-32.

15. Khor, G.H., et al., *Involvement of CELSR3 Hypermethylation in Primary Oral Squamous Cell Carcinoma.* Asian Pac J Cancer Prev, 2016. **17**(1): p. 219-23.

16. *Comprehensive molecular characterization of human colon and rectal cancer.* Nature, 2012. **487**(7407): p. 330-7.

17. Kim, H., et al., *Newly Identified Cancer-Associated Role of Human Neuronal Growth Regulator 1 (NEGR1).* J Cancer, 2014. **5**(7): p. 598-608.

18. Tan, S.X., et al., *Methylation of PRDM2, PRDM5 and PRDM16 genes in lung cancer cells.* Int J Clin Exp Pathol, 2014. **7**(5): p. 2305-11.

19. Shukla, V., et al., *ASXL3 Is a Novel Pluripotency Factor in Human Respiratory Epithelial Cells and a Potential Therapeutic Target in Small Cell Lung Cancer.* Cancer Res, 2017. **77**(22): p. 6267-6281.
